# The role of multimodality imaging in diabetic cardiomyopathy: a brief review

**DOI:** 10.3389/fendo.2024.1405031

**Published:** 2024-12-23

**Authors:** Fadi W. Adel, Horng H. Chen

**Affiliations:** Department of Cardiovascular Medicine, Mayo Clinic, Rochester, MN, United States

**Keywords:** diabetic cardiomyopathy, heart failure, diabetes mellitus, multi-modality imaging, echocardiography, cardiac MRI, cardiac nuclear imaging, diabetes therapeutics

## Abstract

Diabetic cardiomyopathy (DMCM), defined as left ventricular dysfunction in the setting of diabetes mellitus without hypertension, coronary artery disease or valvular heart disease, is a well-recognized entity whose prevalence is certainly predicted to increase alongside the rising incidence and prevalence of diabetes mellitus. The pathophysiology of DMCM stems from hyperglycemia and insulin resistance, resulting in oxidative stress, inflammation, cardiomyocyte death, and fibrosis. These perturbations lead to left ventricular hypertrophy with associated impaired relaxation early in the course of the disease, and eventually culminating in combined systolic and diastolic heart failure. Echocardiography, cardiac nuclear imaging, and cardiac magnetic resonance imaging are crucial in the diagnosis and management of the structural and functional changes associated with DMCM. There appears to be a U-shaped relationship between glycemic control and mortality. Exogenous insulin therapy, while crucial, has been identified as an independent risk factor for worsening cardiovascular outcomes. On the other hand, Glucagon-like Peptide-1 Receptor Agonists and Sodium–Glucose Cotransporter 2 Inhibitors appear to potentially offer glycemic control and cardiovascular protection. In this review, we briefly discuss the pathophysiology, staging, role of multimodality imaging, and therapeutics in DMCM.

## Introduction

In 2023, foremost societies, including the American Heart Association (AHA), the American College of Cardiology (ACC), and the European Society of Cardiology (ESC) endorsed its formal definition (1). Diabetic cardiomyopathy (DMCM) is defined as left ventricle (LV) dysfunction in the presence of diabetes mellitus (DM), whether it is type 1 (DM1) or type 2 (DM2), and in the absence of hypertension (HTN), obstructive epicardial coronary artery disease (CAD), and valvular heart disease (VHD) (2, 3).

Worldwide, the prevalence of DM increased from 151 million in 2000 to 537 million in 2021, and it is projected to increase to 643 million by 2030 (4). Among diabetic patients, the prevalence of DMCM ranges from 16.9% (5) to about 67% (6), depending on the criteria used for definition.

In this review, we will briefly discuss the pathophysiology, staging, and therapeutics, with a dedicated focus on the role of multi-modality imaging.

## Pathophysiology & staging

**Figure 1:**
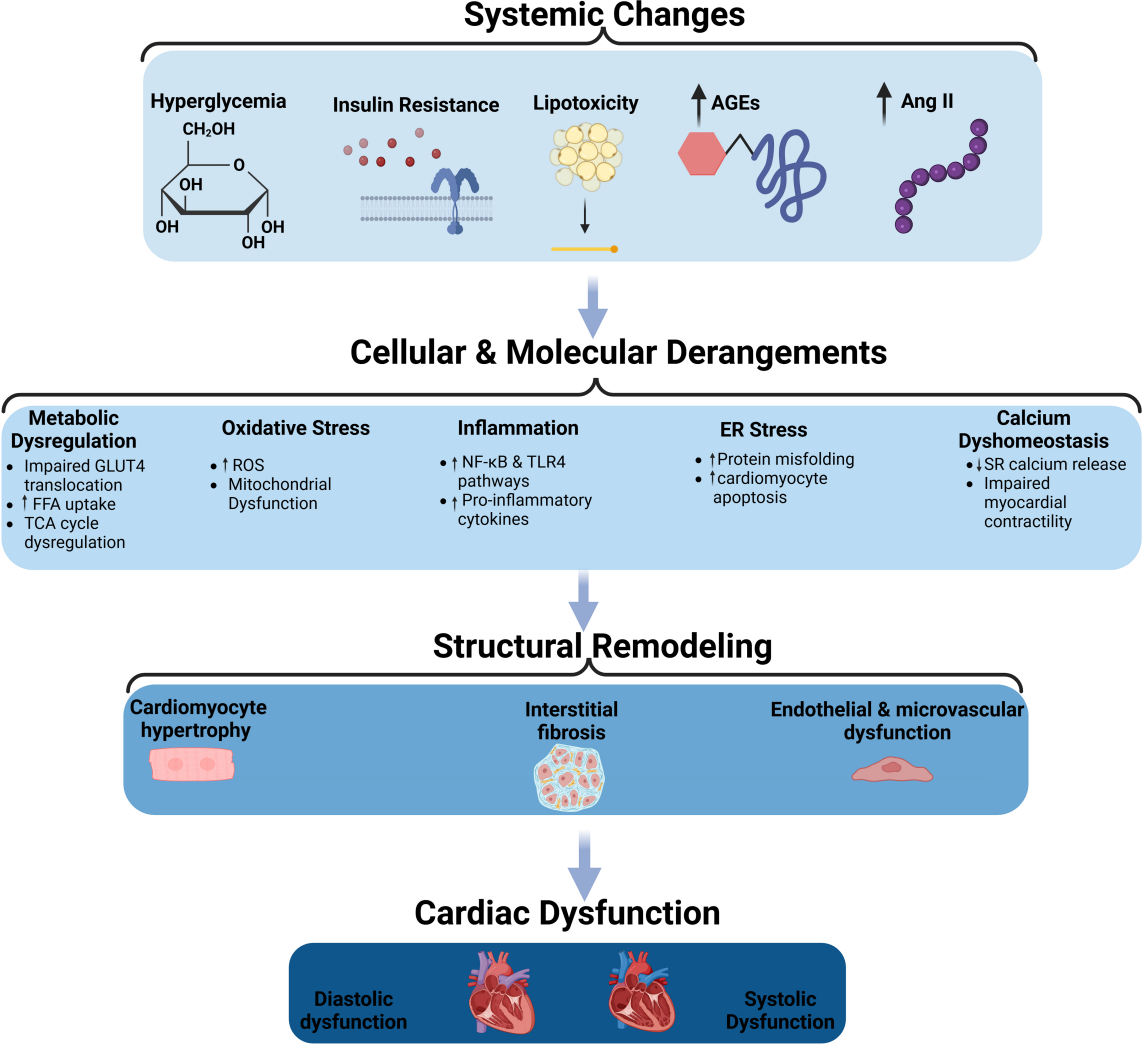
The pathophysiological mechanisms of diabetic cardiomyopathy. Schematic representation of the systemic, cellular, and molecular changes leading to structural remodeling and cardiac dysfunction in diabetic cardiomyopathy. Systemic changes such as hyperglycemia, insulin resistance, lipotoxicity, advanced glycation end-products (AGEs), and increased angiotensin II (Ang II) contribute to cellular and molecular derangements, including metabolic dysregulation, oxidative stress, inflammation, endoplasmic reticulum (ER) stress, and calcium dysregulation. These changes promote structural remodeling characterized by cardiomyocyte hypertrophy, interstitial fibrosis, and endothelial/microvascular dysfunction, ultimately leading to diastolic and systolic cardiac dysfunction. AGEs, Advanced Glycation End-products; Ang II, Angiotensin II; FFA, Free Fatty Acids; GLUT4, Glucose Transporter Type 4; TCA cycle, Tricarboxylic Acid Cycle; ROS, Reactive Oxygen Species; NF-κB, Nuclear Factor Kappa-Light-Chain-Enhancer of Activated B Cells; TLR4, Toll-like Receptor 4; ER, Endoplasmic Reticulum; SR, Sarcoplasmic Reticulum.

The pathophysiology of DMCM involves a complex interplay of insulin resistance mediating hyperglycemia and lipotoxicity (**Figure 1**). In that milieu, oxidative stress ensues, with accompanying inflammation, resulting in cardiomyocyte calcium dyshomeostasis (7, 8), cardiomyocyte death (9, 10) and, later, hypertrophy (11), along with endothelial damage (12, 13) and interstitial fibrosis (14–16).

Early, patients usually experience impaired myocardial relaxation, which manifests as mild diastolic dysfunction (10, 17). As the disease progresses, patients develop left ventricular hypertrophy (LVH) in the setting of cardiomyocyte hypertrophy, interstitial fibrosis, and maladaptive inflammatory response. Clinically, this manifests as more advanced diastolic dysfunction and possibly early systolic dysfunction (10). In the late stages of the disease, severe neurohormonal disturbances, such as activation of both the Angiotensin-II and sympathetic nervous systems (17), lead to significant increases in LV thickness, mass, and size, with an accompanying impairment in both systolic and diastolic function (10, 17, 18).

## Role of multimodality imaging

From a clinical perspective, the diagnosis of DMCM requires the utilization of at least one imaging modality to confirm LV structural and functional impairments. Imaging also aids in monitoring the course of the disease and assess the impact of treatment. Furthermore, different imaging modalities allow the assessment of different mechanisms contributing to the development and progression of DMCM (3, 19, 20).

### Echocardiography

Echocardiography is the gold standard in diagnosing DMCM, owing to its high temporal and spatial resolutions, accessibility, affordability, and harmlessness (19, 21). One of the earliest features of DMCM is the impairment of diastolic function (19). In a case-control study among patients with DM2 with a median duration of > 5 years, systolic function was preserved among all patients. However, 54% of the diabetic patients had diastolic dysfunction, compared to 11% among non-diabetic controls, with more incident diastolic dysfunction correlating with duration of diabetes (22).

Further, Somaratane et al. inspected the prevalence of LVH in a cohort of DM2, and found that 56% of the diabetic population exhibited this structural abnormality. Interestingly, electrocardiograms (EKG) only detected 5% of LVH cases, and while NT-proBNP was superior to EKG, it remained inadequate; this study underscores the significant utility of echocardiography in detecting LVH (23).

Additionally, in patients with DM1 and average HbA1c of 8%, the lateral mitral annular early diastolic velocity was lower compared to non-diabetic controls, which is a marker of diastolic dysfunction (24). Moreover, in DM2 patients with a mean diabetes duration of 6.3 years, 45% demonstrated abnormal global longitudinal strain, which conferred a higher risk for the development of all-cause mortality or hospitalization (25).

Impaired diastolic parameters among diabetic patients are evidently associated with worse clinical outcomes. Rorth et al. showed that, among DM1 patients, higher filling pressures, as measured by early mitral inflow velocity and mitral annular early diastolic velocity ratio (e/e’), was associated with a higher risk of non-fatal myocardial infarction, cerebrovascular accidents, and death (26). Similarly, From et al. demonstrated that, among DM2 patients, the cumulative probability of both the incidence of heart failure and death more than doubled among diabetics with higher filling pressures as measured by e/e’ ratios (27).

In summary, echocardiography remains indispensable in the early detection and monitoring of DMCM, providing critical prognostic information that guides clinical decision-making and management. Its ability to identify subtle changes in cardiac structure and function underscores its role as the gold standard imaging modality in this patient population.

However, this technique is not without its challenges; the diagnostic accuracy can be operator-dependent, and image quality may be compromised in patients with poor acoustic windows, such as those with obesity or chronic lung disease (28). Additionally, while echocardiography is excellent for structural assessment, it provides limited information on myocardial tissue characterization and metabolism, which are crucial for understanding the pathophysiology of DMCM (3).

### Cardiac magnetic resonance imaging

Cardiac magnetic resonance imaging (CMR) possesses higher spatial and temporal resolution than echocardiography, in addition to allowing the assessment of myocardial fibrosis and altered metabolism, which are hallmarks of DMCM (3, 19, 21).

Among DM1 patients, average HbA1c levels were inversely associated with stroke volume and positively correlated with the presence of myocardial scar tissue (29). Additionally, myocardial perfusion reserve and diastolic strain rate were abnormal in diabetic patients when compared to controls; further, increased myocardial triglyceride content, but not impaired myocardial flow reserve, was associated with abnormal diastolic function, suggesting that steatosis, but not necessarily small vessel disease, may be responsible for early diastolic dysfunction in DMCM (30). In a cohort derived from the general population, CMR revealed that pre-DM and DM were associated with LV remodeling compared to non-diabetic controls (31). Fibrotic tissue, as determined by late gadolinium enhancement, can be easily assessed using CMR and it has been associated with worse major adverse cardiovascular events (MACE) in diabetic patients (32).

However, CMR remains underutilized, largely owing to its perceived expense burden, patient comfort issues, and longer examination times (33). Additionally, traditional, older generation Gadolinium-based contrast media (CGBM) were associated with a low but serious risk of nephrogenic systemic fibrosis. Thankfully, with group II CGMB, this risk is significantly diminished (34). With further advancements in imaging and technology, it may be feasible in the future to incorporate CMR as a cornerstone in DMCM management.

### Nuclear imaging

Nuclear imaging allows the detection of low-density processes, making it possible to measure myocardial metabolism and to assess molecular imaging (20). Gated SPECT possesses the capability to assess myocardial perfusion and LV function; however, limited data exist on its widespread clinical utility in DMCM (3).

Positron emission tomography (PET) allows flexibility in radiotracer design radiolabeled with a variety of radionuclides, and radiotracers administered at low doses do not alter metabolic processes (20). In order to circumvent the low spatial resolution observed in PET, it is usually combined with computer tomography or CMR to allow for accurate radiotracer localization (3, 20).

PET imaging has been utilized to study a multitude of metabolic parameters in DMCM. Using PET-CMR in DM2 patients with average HbA1c of 7.1% and mean diabetes duration of 4 years, Rijzewijk et al. demonstrated impaired diastolic parameters among diabetics; more importantly, they revealed increased myocardial fatty acid uptake and oxidation compared to controls, indicating myocardial metabolic remodeling (35). Similarly, in DM1 patients, PET imaging revealed increased free fatty acid utilization and decreased myocardial glucose uptake (36). Further, phase analysis of gated SPECT MPI revealed that asymptomatic DM2 patients with normal perfusion scans exhibited significant left ventricular mechanical dyssynchrony, particularly in those with a diabetes duration of more than 15 years (37). In a study investigating the association between diabetes mellitus and myocardial glucose uptake using 18F-FDG PET/CT, it was demonstrated that DM is significantly associated with decreased myocardial glucose metabolism, with up to 84% of diabetic patients showing poor FDG uptake. Furthermore, multivariate logistic regression analysis revealed that gender (male), Homeostatic Model Assessment of Insulin Resistance, and metabolic dysfunction-associated steatotic liver disease were independent risk factors for poor myocardial FDG uptake in diabetic patients (38).

In summary, nuclear imaging techniques like PET and gated SPECT are valuable in assessing myocardial metabolism and function in DMCM, revealing significant alterations such as impaired diastolic parameters, increased myocardial fatty acid uptake and oxidation, decreased myocardial glucose uptake, and left ventricular mechanical dyssynchrony, particularly in patients with prolonged diabetes duration.

The main challenges associated with nuclear imaging include its high cost and limited availability (20). Additionally, the relatively low spatial resolution of PET compared to CMR can limit its ability to detect small areas of myocardial scar or fibrosis (19). The use of ionizing radiation in both PET and SPECT raises concerns about radiation exposure, particularly in younger patients and those requiring repeated imaging studies (20). Furthermore, the interpretation of nuclear imaging studies requires specialized expertise, which may not be readily available in all clinical settings (3).

In conclusion, while each imaging modality offers unique benefits in the assessment of DMCM, they also come with specific challenges (**Table 1**). A multimodality approach, leveraging the strengths of each technique, can provide a comprehensive evaluation of diabetic cardiomyopathy, improving diagnostic accuracy and informing therapeutic strategies. Future advancements in imaging technology and reductions in cost may further enhance the integration of these modalities into routine clinical practice.

**Table 1:** Strengths and limitations of multimodality imaging techniques in diabetic cardiomyopathy.

Modality	Strength	Limitation
Echocardiography	• High temporal and spatial resolutions, accessibility, affordability, harmlessness • Effective in detecting early diastolic dysfunction • Critical for early detection and monitoring of DMCM	• Operator-dependent diagnostic accuracy • Compromised image quality in patients with poor acoustic windows • Limited information on myocardial tissue characterization and metabolism
Cardiac MRI	• Higher spatial and temporal resolution, assesses myocardial fibrosis and altered metabolism • Can identify myocardial scar tissue and abnormal diastolic function • Comprehensive tissue characterization, high-resolution imaging	• Perceived expense burden, patient comfort issues, longer examination times • Risk of nephrogenic systemic fibrosis with older generation Gadolinium-based contrast media
Nuclear Imaging	• Detects low-density processes, measures myocardial metabolism, assesses molecular imaging • Flexible radiotracer design, accurate radiotracer localization with combined techniques • Reveals significant metabolic alterations in DMCM	• High cost and limited availability • Low spatial resolution compared to CMR, concerns about radiation exposure • Requires specialized expertise for interpretation

## Therapeutics

In the UK Prospective Diabetes Study (UKPDS), a 1% decrease in HbA1c was associated with a 16% decrease in risk of myocardial infarction (39). Yet, it has become apparent that there is a U-curve relationship between HbA1c and mortality in diabetic patients with heart failure (40). Indeed, intensive glycemic control did not reduce cardiovascular events, and it was associated with a 47% increase in incident heart failure (41). Exogenous insulin therapy, which is utilized by all DM1 patients and 1/3^rd^ of DM2 with heart failure patients (42), has been shown to be a risk factor for incident heart failure (43). Similarly, exogenous insulin use is associated with a higher risk of all-cause mortality, and HF rehospitalization (44). In a preclinical rodent model of experimental DM, insulin use was associated with increased interstitial fibrosis and cardiomyocyte apoptosis compared to both non-diabetic and untreated diabetic controls, further supporting the hypothesis that long-term exogenous insulin may adversely affect the myocardium (45).

Metformin use has yielded inconsistent results. In a metanalysis including 13,110 DM patients, metformin use did not bestow any HF benefit (46). On the other hand, in the UKPDS, metformin use was associated with a 39% reduction in the risk of myocardial infarction (47).

Sulfonylureas and thiazolidinediones have been associated with an increase in all-cause mortality and/or HF hospitalization among DM2 patients (48, 49). Dipeptidyl Peptidase 4 Inhibitors (DPP-4i) have not been associated with any cardiovascular benefit. In fact, saxagliptin has been associated with an increased risk of heart failure hospitalization (50). In the American Diabetes Association (ADA) Consensus Report in 2022, DPP-4i should be avoided in DM patients with ACC/AHA stage B and C (51). Glucagon-like Peptide-1 Receptor Agonists (GLP-1 RAs) use has been associated with a decreased risk of MACE in DM2 patients with established cardiovascular disease (52). While randomized clinical trials for GLP-1 RAs have shown no benefit when it comes to heart failure (53–56), the HARMONY Outcomes trial suggested a 29% reduction in heart failure hospitalization as a secondary outcome (57); further, in one meta-analysis, there was a 9% reduction in heart failure hospitalization (58). In STEP-HFpEF, heart failure hospitalization, as an exploratory end-point, seemed to be lower in the semaglutide group compared to placebo (59).

Sodium–Glucose Cotransporter 2 Inhibitors (SGLT2i) have been shown to decrease the risk of MACE and heart failure hospitalization among diabetics (60, 61). In fact, the ADA recommends SGLT2i use among DM patients with heart disease (62).

## Conclusion

In conclusion, DMCM is a major and increasing health concern, fueled by the global rise in DM incidence (4–6). The complex pathophysiology of DMCM, characterized by insulin resistance, hyperglycemia, and lipotoxicity (18), leads to oxidative stress, inflammation, cardiomyocyte death, and fibrosis. These processes result in LVH and dysfunction (7–14). Multimodality imaging, encompassing echocardiography, CMR, and nuclear imaging, is crucial for the diagnosis, staging, and management of DMCM. Each imaging modality provides distinct insights into cardiac structure and function, metabolic changes, and tissue characterization, thus enhancing our comprehension and management of this intricate condition.
